# GMP production of [^18^F]FE-PE2I on a TRACERLab FX2 N synthesis module, a radiotracer for in vivo PET imaging of the dopamine transport

**DOI:** 10.1186/s41181-024-00269-9

**Published:** 2024-05-02

**Authors:** Mélodie Ferrat, Mohammad M. Moein, Carmen Cananau, Tetyana Tegnebratt, Paul Saliba, Fredrik Norman, Carsten Steiger, Klas Bratteby, Erik Samén, Kenneth Dahl, Thuy A. Tran

**Affiliations:** 1 Department of Radiopharmacy, Karolinska University Hospital, 171 76 Stockholm, Sweden; 2 Department of Oncology and Pathology, Karolinska Institutet, 171 76 Stockholm, Sweden; 3 Department of Medical Radiation Physics and Nuclear Medicine, Karolinska University Hospital, 171 76 Stockholm, Sweden

**Keywords:** [^18^F]FE-PE2I, GE TRACERLab FX2 N, Automation, Dopamine transporter** (**DAT), Fluorine-18, PET, Radiochemistry, GMP

## Abstract

**Background:**

Parkinson's disease is a neurodegenerative disorder that is characterized by a degeneration of the dopaminergic system. Dopamine transporter (DAT) positron emission tomography (PET) imaging has emerged as a powerful and non-invasive method to quantify dopaminergic function in the living brain. The PET radioligand, [^18^F]FE-PE2I, a cocaine chemical derivative, has shown promising properties for in vivo PET imaging of DAT, including high affinity and selectivity for DAT, excellent brain permeability, and favorable metabolism. The aim of the current study was to scale up the production of [^18^F]FE-PE2I to fulfil the increasing clinical demand for this tracer.

**Results:**

Thus, a fully automated and GMP-compliant production procedure has been developed using a commercially available radiosynthesis module GE TRACERLab FX2 N. [^18^F]FE-PE2I was produced with a radiochemical yield of 39 ± 8% (n = 4, relative [^18^F]F^−^ delivered to the module). The synthesis time was 70 min, and the molar activity was 925.3 ± 763 GBq/µmol (250 ± 20 Ci/µmol). The produced [^18^F]FE-PE2I was stable over 6 h at room temperature.

**Conclusion:**

The protocol reliably provides a sterile and pyrogen–free GMP-compliant product.

## Background

The dopamine transporter (DAT) is a plasma membrane protein expressed exclusively on presynaptic dopaminergic neurons in the central nervous system (CNS). It is responsible for regulating the synaptic concentration of dopamine out of the synaptic cleft into the neurons. DAT imaging in the nigrostriatal system is a well-established tool for the evaluation of dopaminergic function in neurodegenerative disorders, e.g., Parkinson's disease (PD) and Parkinson´s plus-syndromes or the atypical parkinsonians (APS) (Palermo and Ceravolo 2019; Varrone and Halldin 2010).

Until now, DAT imaging has predominantly been performed on a daily clinical practice using the commercially available SPECT (single-photon emission computed tomography) radiopharmaceutical, [^123^I]FP-CIT ([^123^I]-ioflupane, DaTSCAN, GE HealthCare), an approved tracer for PD diagnostics (Darcourt et al. 2010). Moreover, FP-CIT SPECT is also being used to differentiate neurodegenerative disorders from essential tremor, drug-induced or vascular forms of parkinsonism but cannot differentiate between PD and APS.

Positron emission tomography (PET) imaging, on the other hand, is a more sensitive technique to measure the density and activity of DAT in the brain, which could potentially be useful for diagnosis and evaluation of possible treatments (Jakobson Mo et al. 2018). Several radioligands for imaging DAT have been reported and applied for PET in human subjects, most of which have been derivatives of cocaine, [^11^C]PE2I (Halldin et al. 2003), [^11^C]β-CIT (Müller et al. 1993), [^11^C]β-CIT-FE (Halldin et al. 1996), [^18^F]β-CFT (Laakso et al. 1998), [^18^F]FECNT (Goodman et al. 2000), and [^18^F]LBT-999 (Varrone et al. 2011). The ^18^F-labelled analogue of PE2I, (E)-N-(3-iodoprop-2-enyl)-2β-carbofluoroethoxy-3β-(4’-methyl-phenyl) nortropane ([^18^F]FE-PE2I), has shown excellent properties for in vivo imaging of DAT, which includes, high affinity and selectivity, excellent brain permeability, favorable metabolism, and shows appropriate in vivo kinetics (Schou et al. 2009; Varrone et al. 2012; Sasaki et al. 2011). The high affinity of [^18^F]FE-PE2I even allows the visualization and quantification of ligand binding to DAT in the substantia nigra.

**Scheme 1:**
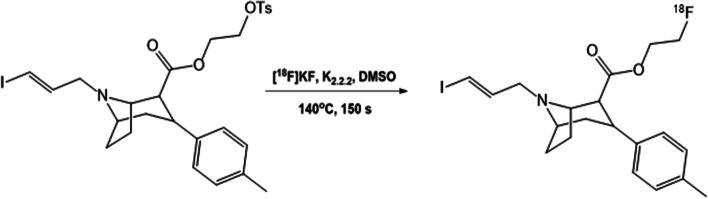
Radiosynthesis of [^18^F]FE-PE2I

The synthesis of [^18^F]FE-PE2I was first described by Schou et al., via a two-step and two-pot procedure. Although the yield and purity of [^18^F]FE-PE2I (RCY of 7% and a RCP > 95%) was sufficient for the initial Non-Human Primate (NHP) PET evaluation, the synthesis procedure was deemed unsuitable for routine clinical productions (Schou et al. 2009; Varrone et al. 2012). Therefore, a simplified, one-step radiofluorination procedure was later presented by the same research group (Scheme 1) using the K_2_CO_3_/K_222_ elution method. This semi-automated method provided [^18^F]FE-PE2I in good and reproducible yields (RCY = 20%) and high radiochemical purity (RCP > 98% (n = 4)) (Stepanov et al. 2012). This method has a high potential to be implemented for automation in human PET applications.

Although the automation of this one-step procedure appears to be straightforward, scaling [^18^F]FE-PE2I synthesis might be challenging due to the presence of radiolysis observed during labeling, purification and formulation steps. The precursor is sensitive due to possible degradation when facing harsh eluent (K_2_CO_3_/K_222_). The optimization of the elution condition was performed by Bratteby et al. using Bu_4_NH_2_PO_4_, which shows a high isolated RCY of up to 62% for small activity synthesis under 40 GBq. When scaling up the synthesis over 40 GBq, auto-radiolysis has been observed, which leads to a low RCY (Bratteby et al. 2021). [^18^F]FE-PE2I has been tested with a RCY of approximately 40% using Bu_4_NH_2_PO_4_ elution with a starting activity of 80 GBq. However, this method has not been implemented for good manufacturing procedures (GMP) use (Bratteby et al. 2021).

Moreover, [^18^F]FE-PE2I is clinically produced via this one-step synthesis method using a GMP-automated cassette-based radiochemistry module (Synthera®^+^, IBA) with a preparative HPLC system. The production of [^18^F]FE-PE2I provides a RCY of 35% (10.5 GBq) with a starting activity of 45 GBq (Bratteby et al. 2021).

The goal of the current work was to enable large-scale production of [^18^F]FE-PE2I to meet the increasing clinical demand for an ^18^F-labelled DAT PET imaging agent. Herein, we report the fully automated radiosynthesis of [^18^F]FE-PE2I performed under GMP conditions using a commercial radiofluorination module (GE TRACERLab FX2 N) and its comprehensive validation for clinical routine human use.

## Methods

### Experimental and materials

All chemicals and reagents were obtained from Sigma-Aldrich and were used as received without further purification. The precursor tosylethyl-PE2I was purchased from Pharmasynth AS (Estonia). Solid-phase extraction cartridges: Sep-Pak Accell Plus QMA Plus Light Cartridge and tC18 Plus short Cartridge were purchased from Waters Corporation (Milford, Massachusetts, USA).

High-performance liquid chromatography (HPLC) analysis of compounds was performed on a Poroshell 120 EC C-18, 3 × 150 mm, i.d. 2.7 µm column on an Agilent 1260 HPLC system (UV absorbance 220 nm) using TFA 0,1%/ACN as eluent (gradient elution) with a flow of 0.5 mL/min. This quality control is performed pre-release on all batches. For more information, please refer to the section “Quality control procedure”.

The purification of [^18^F]FE-PE2I is performed with a semi preparative ACE HPLC column (5 μm C-18 HL, 10 × 250 nm, Advanced Chromatography Technologies) using ACN:H_2_O:TFA 175:325:0.5 (v/v/v) mobile phase (isocratic elution). The HPLC purification system consists of a pump (Sykom), an automated sample injection equipped with a 5 mL stainless-steel loop. UV detector from Knauer and a gamma radioactivity PIN diode detector.

Radio-thin layer chromatography (radio-TLC) analyses were run on TLC Silica gel 60 F_254_, glass plates, 2.5 × 7.5 cm (Merck) (stationary phase) using acetonitrile and 0.1 M citrate buffer, pH 5.0 (1:1, v/v) as a mobile phase. The TLC analytical method set-up: scan speed 1 mm/s; scan length 0–7.5 cm; sample volume 1–2 µL; minimum 200,000 counts acquired. The method allows determination of the retardation factor (Rf) and quantification of radiochemical purity for each component. Radioactivity spots were detected using an automatic radio-TLC scanner (Scan-RAM™ PET/SPECT radio-TLC scanner). This quality control is performed pre-release on all batches. For more information, please refer to the section “Quality control procedure”.

The Gas chromatography (GC) method is developed for a 30 m long Res-Solv capillary column having 0.53 mm inner diameter and a 1.0 µm film. The flame ionization detector (FID) is used as detector to analyze ions formed during combustion of organic compound in synthetic air and hydrogen gas. The injection volume was 2 µL.

The GC analytical method: the split ratio was 1:80 and the inlet and detector temperature were 250 °C. The temperature program: 35 °C for 3.5 min after injection, ramp to 240 °C at 70 °C/min, hold at 240 °C for 3 min, cool to 35 °C. The GC is performed to verify a separation between solvents to be analyzed, i.e. DMSO, acetonitrile and ethanol. This quality control is performed pre-or post-release on all batches. For more information, please refer to the section “Quality control procedure”.

The synthesis method sequence for GE TRACERLab FX2 N system was developed in-house at Karolinska Radiopharmacy department, Karolinska University Hospital. Production of [^18^F]FE-PE2I was performed in a class C cleanroom laboratory and the GE TRACERLab FX2 N synthesizer is located in a BBS hotcell (Comecer). Two product vials are assembled in a laminar airflow workbench with a sterile product filter and a ventilation filter to receive the radiolabeled tracer.

The product is finally released by an onsite QA/QP prior to use in human PET studies.

[^18^F]FE-PE2I was approved by the Swedish Medical Product Agency for clinical examinations in patients on a yearly license. PET/CT imaging was performed on a GE Discovery MI PET/CT (GE Healthcare, Milwaukee, WI).

Patients received a dose of 200 MBq [^18^F]FE-PE2I bolus intravenous injection and then allowed to rest for 30 min before being placed supine and head-first, in the PET/CT scanner. A low-dose CT for attenuation correction was performed (100 kV, 0 mAs, slice thickness of 3.75 and FOV: 700 mm), before a static PET acquisition acquired in list-mode for 12 min. PET data were reconstructed by the ordered-subsets expectation maximization (OSEM) algorithm (3 iterations, 34 subsets) and a 3 mm Gauss filter, after application of all suitable corrections such as those for photon attenuation, scattered radiation, time-of-flight (TOF), point spread function (PSF) and radioactive decay of the [^18^F]FE-PE2I.

For the visual assessment and image interpretation, the reconstructed PET-data were analyzed using the commercially available Hermes software (Hermes Medical Solution, Sweden) (https://www.hermesmedical.com/our-software/).

## Results

### Synthesis of [^18^F]FE-PE2I

**Fig. 1:**
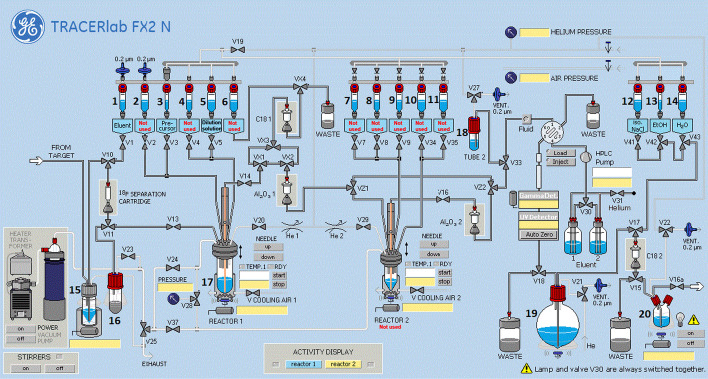
Schematic diagram of GE TRACERlab FX2 N module used for synthesis of [^18^F]FE-PE2I

**Fig. 2:**
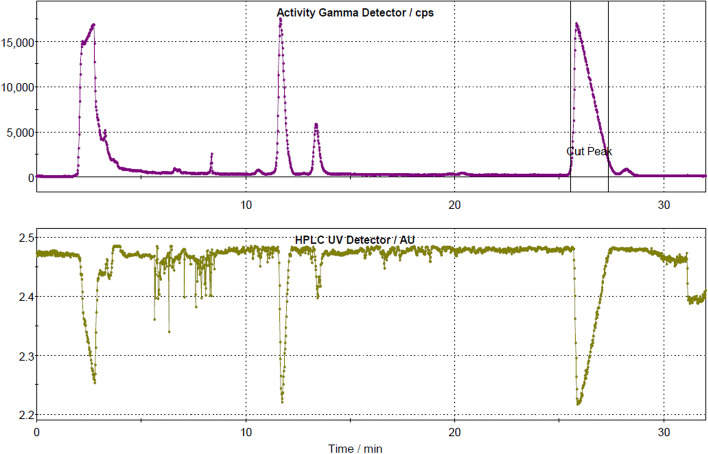
A typical semi-preparative HPLC chromatogram of [^18^FE]FE-PE2I Solution for Injection

A schematic diagram of the GE TRACERLab FX2 N radiosynthesis module used for the synthesis of [^18^F]FE-PE2I is shown in Fig. 1. The in-house developed reaction sequence for [^18^F]FE-PE2I (Scheme 1) involves three main steps: (1) the initial azeotropic drying of [^18^F]F^−^; followed by (2) nucleophilic fluorination of the precursor compound; and finally (3) HPLC purification and formulation of the final product. The synthesis module was operated in the following sequences with numerical references to vials (**1**–**20**) in Fig. 1:

1. At the end of bombardment, aqueous [^18^F]fluoride ([^18^F]F^−^, ~ 50–83 GBq) was produced following the nuclear reaction: ^18^O(p, n)^18^F using a General Electric Medical PETtrace 800 cyclotron (16.4 MeV). The short-lived radionuclide was transferred from the target to a collection vial **15** via a stream of helium gas (6.0, AGA).
2. The aqueous [^18^F]F^−^ solution was transferred from the collection vial **15** via V10 – 11 over a pre-activated (10 mL 0.5 M potassium carbonate and 10 mL > 16 MΩ water) Sep-Pak Accell Plus QMA Plus Light Cartridge (Waters). [^18^F]F^−^ was quantitively trapped on the QMA cartridge and [^18^O]H_2_O was recovered in a recovery vial **16**.
3. The trapped [^18^F]F^−^ (~ 50–83 GBq) are eluted from the QMA using 1.0 mL of a Kryptofix eluting solution (4.7 mg Kryptofix®222, 0.9 mg K_2_CO_3_, 40 µL > 16 MΩ water, 960 µL acetonitrile), preloaded into vial **1** and delivered to the reaction vial **17**.
4. The [^18^F]F^−^ mixture in vial **17** was first dried azeotropically at 85 °C under N_2_ flow and vacuum for 7 min, and later at 110 °C under N_2_ flow and vacuum for another 5 min. The reaction vial was then cooled to 60 °C prior to the next step.
5. The precursor solution (1.0 mg tosylethyl-PE2I dissolved in 1.5 mL DMSO) is preloaded into vial **3** and further added to the reaction vial **17**. The reactor is sealed and heated to 140 °C for 150 s. After completed reaction, the reactor was cooled to 60 °C prior to the next step.
6. The crude reaction mixture was then diluted with dilution solution containing 1.5 mL of mobile phase (35:65, acetonitrile:0.1% trifluoracetic acid with 0.5 mg/mL sodium ascorbate) and 2.0 mL sodium ascorbate solution (5 mg/mL in sterile water), which was added from vial **5** to the reaction vessel **17,** prior to HPLC purification.
7. The content of vial **17** was first transferred into an intermediate vial **18**, before it was delivered to the HPLC loop (5 mL) via a fluid detector. The solution was further injected into a semi-preparative HPLC column (ACE 5, C18-HL, 250 × 10 mm i.d., 5 µm), and eluted with mobile phase (35:65 acetonitrile: 0.1% trifluoracetic acid with 0.5 mg/mL sodium ascorbate) at a flow rate of 5 mL/min. The elute was monitored by UV (λ = 254 nm), and a radioactivity detector connected in series.
8. A typical semi-preparative HPLC chromatogram is displayed in Fig. 2 using an isocratic elution of 35:65 acetonitrile: 0.1% trifluoracetic acid with 0.5 mg/mL sodium ascorbate at a flow rate of 5 mL/min, and a semi-preparative column ACE 5 C18-HL, 250 × 10 mm i.d., 5 µm. The fraction containing the desired product, [^18^F]FE-PE2I (retention time ≈ 25 min), was collected into a collection vessel **19**, which was preloaded with 40 mL sodium ascorbate solution (5 mg/mL in sterile water).
9. The resulting solution is then transferred via V17 and V15 over a pre-activated (10 mL ethanol 99.5% and 10 mL of sodium ascorbate solution (5 mg/mL in sterile water)) Sep-Pak tC18 Plus short Cartridge (Waters). [^18^F]FE-PE2I was trapped on the tC18 cartridge and immediately thereafter washed with 10 mL sodium ascorbate solution (5 mg/mL in sterile water), preloaded into vial **14**.
10. [^18^F]FE-PE2I was eluted using 1.5 mL ethanol, preloaded into vial **13** and delivered to final mixing vial (**20**) which had been preloaded with 233 mg sodium ascorbate dissolved in 10 mL of saline (0.9% NaCl, pH 4.5–7.0). The final solution further diluted with 117 mg sodium ascorbate in saline from vial **12**.
11. Finally, the formulated product (volume = 16.5 mL, ~ 9% ethanol in saline) was delivered into two separate product vials via two different sterile filters (0.22 µm sterile Millex-GV filter, Millipore). The final volume obtained for product vial 1 and product vial 2 was 8 mL and 6 mL, respectively. This was accomplished by applying a constant helium pressure (1 Bar) to the final mixing vial **20** for a specific timeframe (product vial 1 = 50 s; product vial 2 = 40 s). In our setup, with 1 Bar input pressure and a tube length of approximately 0.5 m, a flow rate of ~ 10 mL/min was generated. The bulk volume of [^18^F]FE-PE2I was then dispensed in two different vials at Karolinska University Hospital with the intention of being transported to and used at different PET/CT units located at different allocations within Stockholm region, as well as to other Nuclear Medicine department due to its valuable half-life of ^18^F (110 min).

### Quality control procedure

The quality control of [^18^F]FE-PE2I Solution for Injection was performed using validated analytical methods based on earlier specifications used in clinical trials at Karolinska Institutet. The specifications, tests and frequency used are described in below and in Tables 1 and 2. The specifications for the standard parameters such as sterility, endotoxins, limits of residual solvents and similar are based on Ph.Eur. The specifications for the chemical impurities, in this case, the mass limit of FE-PE2I and un-identified impurities (in the UV) were justified in earlier studies (Halldin et al. 2003; Sasaki et al. 2011; Bratteby et al. 2021; Fazio et al. 2015), based on single-dose toxicity data, and with regards to genotoxicity studies threshold of 1.5 µg/day according to EMA guidelines.

**Table 1:** Specifications for [^18^F]FE-PE2I Solution for Injection

Parameters	Product specifications	Equipment
Appearance	Clear or slightly yellow. Free of particles	Visual inspection
Filter integrity	≥ 3.5 bar	Bubble point tester
pH	4.5–8.0	pH-meter or pH indicator
Product identity [^18^F]FE-PE2I	|Rt_RD_ – Rt_UV_|≤ 60 s	HPLC
Chemical purity FE-PE2I	Mass limit ≤ 5 µg/patient dose*	
Chemical impurities	Mass limit ≤ 5 µg /patient dose*	
Radiochemical impurity Impurity = B [^18^F]fluoride	≤ 5%	TLC
Total radiochemical purity [^18^F]FE-PE2I RCP_Tot_ = (100 – B) × T	≥ 93%	HPLC and TLC
Mass limit	≤ 5 µg FE-PE2I and ≤ 5 µg impurities per 200 MBq (patient dose)	HPLC
Residual Kryptofix 222 content	< 0.14 mg/mL	Spot test
Bacterial endotoxins	< 11.5 IU/mL	Endosafe
Acetonitrile	< 0.27 mg/mL	GC
DMSO	< 3.3 mg/mL	
Ethanol	< 80 mg/mL	
Sterility	Sterile, 0 CFU	Direct inoculation by an approved contractor
Radionuclidic purity	Principle peak at 511 keV, possible summation peak 1022 keV. Not more than 0.1% radioactivity from total, determined 24 h after EOS	HPGe detector
Radionuclidic identity Half-life [^18^F]	105–115 min	Dose calibrator
Radiochemical stability	RCP_Tot_ ≥ 93% up to 6 h EOS	HPLC
Shelf-life	Batch specific, calculated time where a patient dose exceeds mass limit	HPLC

*A patient dose is defined as 200 ± 20 MBq (for a 70 kg patient) with a mass limit of ≤ 5 µg/patient dose of FE-PE2I and ≤ 5 µg/patient dose of impurities, calibrated at the time of injection

**Table 2:** QC method validation results of [^18^F]FE-PE2I

Parameters	Sample	Acceptance criteria	Results
Specificity (HPLC)	Matrix spiked with FE-PE2I, D-PE2I and Prec-PE2I to get final ⁓ 5 μg/mL	Concentration of FE-PE2I in spiked matrix differ ± 10% of concentration of corresponding peak in SST/Ref Rt (Conc. of spiked matrix ⁓ 5 μg/mL–matrix sample Conc.)/ Conc. of SST ⁓ 5 μg/mL)	0.2% deviation observed
	Sample matrix	No peaks ≥ 60 mAU in the range 6–13 min in the UV	No peak detected
	Sample matrix spiked with [^18^F]^−^	[^18^F]^−^ should elute only in the void peak ⁓ 1–2 min in the RD	Eluted in void volume
	[^18^F]FE-PE2I	Difference in Rt between [^18^F]FE-PE2I (radio chromatogram) and Rt FE-PE2I (SST/Ref UV-chromatogram) < 60 s	5 s
	Ascorbate ID	Rt of ascorbate ⁓ 1:30–2:30 min in the UV chromatogram	Yes
Linearity on UV (HPLC), LOQ, LOD	FE-PE2I	Conc. of FE-PE2I in at least 5 levels in the range ⁓ 0.5–10 μg/mL Linear regression should give a R^2^ ≥ 0.98 Carry-over: The FE-PE2I Conc. in blank after the 10 μg/mL sample should not exceed 10% of Conc. in the previous injection	⁓ 0.5–10 µg/mL LOQ = 0.67 µg/mL LOD = 0.22 µg/mL The coefficients of determination (R^2^) in the aqueous sample were R^2^ = 0.9996
Repeatability, precision (HPLC)	FE-PE2I	The repeatability is tested in the range ⁓ 0.5–10 μg/mL FE-PE2I. The relative response as compared with the SST/Ref, should show an RSD% ≤ 10% on each concentration level (3 injections) and for all injections (9 injections) within the range ⁓ 0.5–10 μg/mL (3 concentrations/3 replicates) Relative response = (Conc.)Sample/(Conc.)SST/Ref	For all tests ≤ 3.53%
Intermediate precision reproducibility (HPLC)	FE-PE2I	On 3 occasions 3 triplicates on 3 concentrations are performed. Different days or analysts, at least two columns from different lots. The relative response, as compared with the SST/Ref, should show an RSD% ≤ 10% on each concentration level (9 injections) and for each analysis (9 injections)	For all tested points ≤ 3.66%
Accuracy (HPLC)	N/A	Accuracy can be inferred once precision, linearity and specificity has been established. Accuracy should be shown in the range ⁓ 0.5–10 μg/mL	Average for 3 occasions 3 triplicates on 3 concentrations are 3,74%
Linearity of radiodetector (HPLC)	[^18^F]FE-PE2I	Linearity in the range of 100–250 MBq/mL considering the radio peak height	0.1–0.25 GBq/mL LOQ = 2.8 MBq/mL LOD = 0.93 MBq/mL
TLC method verification: Specificity	[^18^F]fluoride, [^18^F]FE-PE2I, [^18^F]FE-PE2I spiked with 5% [^18^F]fluoride	The radioactivity of [^18^F]fluoride must be ≤ 5% of the total radioactivity to be released	0.27%
		Retardation factors (Rf) for [^18^F]fluoride = 0–0.1 and for [^18^F]FE-PE2I = 0.7–0.9	[^18^F]fluoride 0–0.04 [^18^F]FE-PE2I 0.832–0.892
		The peak corresponding to [^18^F]fluoride must be completely separated from the peak corresponding to [^18^F]FE-PE2I	[^18^F]fluoride and [^18^F]FE-PE2I were clearly separated, and the method was able to measure a [^18^F]FE-PE2I product spiked with 5%[^18^F]fluoride

#### Appearance

[^18^F]FE-PE2I is visually inspected for its clarity and the absence of visible particles after sufficient radioactive decay to limit high radiation dose to personnel. This quality control is performed on validation and verification batches only.

#### Filter integrity

Filter integrity is determined by using a bubble point test (in-house equipment: 010105280602-A, DM Automation). This quality control is performed pre-release on all batches.

#### pH

The pH of [^18^F]FE-PE2I Solution for Injection is determined by using pH indicator strips 2.0-9.0 (VWR) or pH-meter (type 913, version 2.913.0210, Metrohm). This quality control is performed pre-release on all batches.

#### Radiochemical identity (product identification)

Radiochemical product identity is determined by comparison of a sample from the formulated [^18^F]FE-PE2I solution with a reference solution of [^19^F]FE-PE2I pre-analysed using a high performance liquid chromatography. The eluent is monitored by a UV detector and a radiation detector placed in series. This quality control is performed pre-release on all batches.

#### Chemical purity

**Fig. 3:**
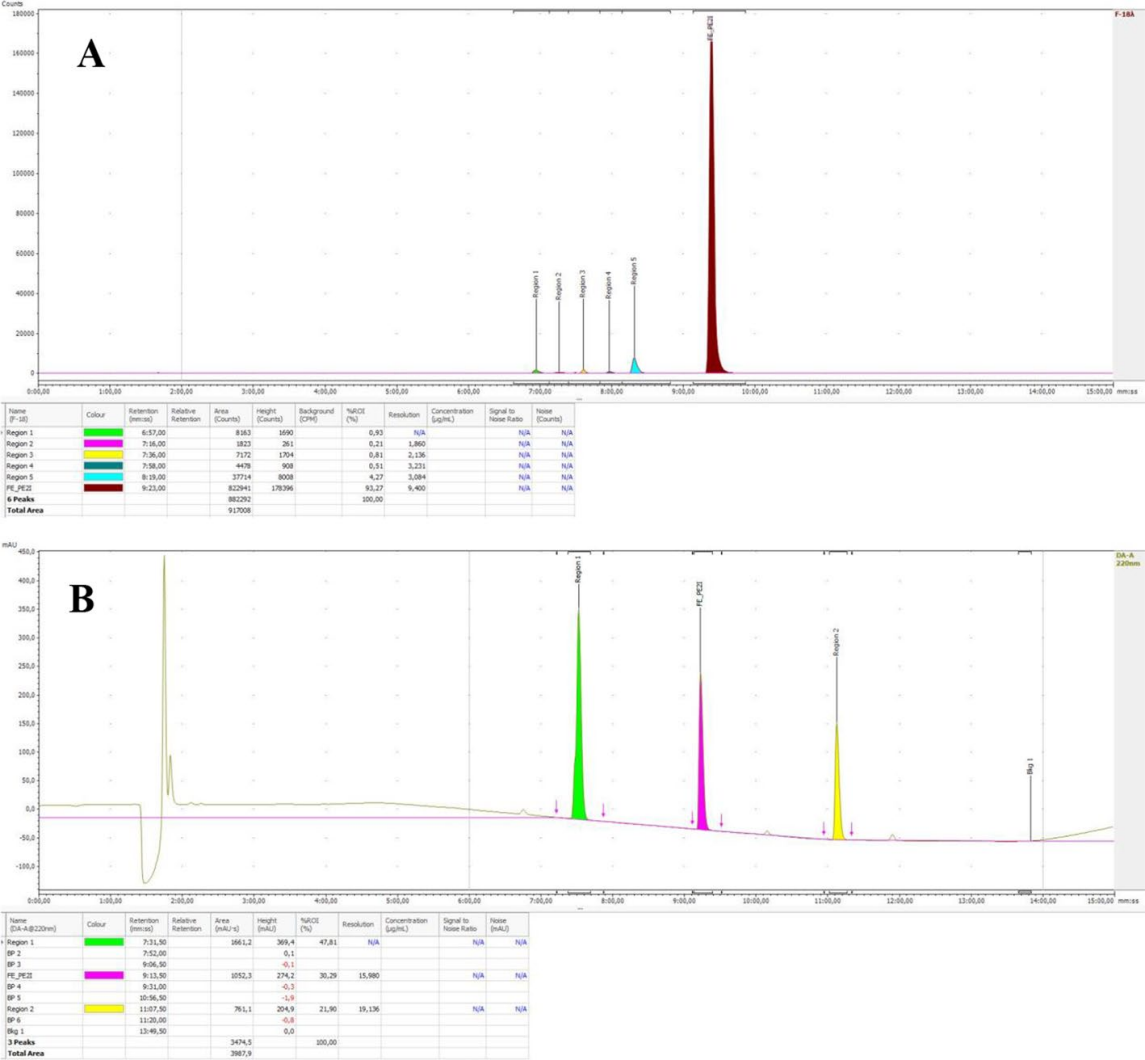
**A:** Radio-chromatogram of [^18^F]FE-PE2I and **B:** FE-PE2I QC SST HPLC chromatogram (5 µg/mL). Region 1: Desmethyl-PE2I, FE-PE2I and Region 2: Tosylethyl-PE2I

The chemical purity of [^18^F]FE-PE2I is estimated from the UV peaks corresponding to [^19^F]FE-PE2I by HPLC analysis (Fig. 3). The HPLC system suitability test (SST) was performed using a 5 µg/mL of a solution mixture containing FE-PE2I, Desmethyl-PE2I (potential by-product) and Tosylethyl-PE2I (precursor) with the resolution (Rs) between D-PE2I (region 1), FE-PE2I and PE2I precursor (region 2) that should be over 2. The results showed Rs over 2 for all the analyzed batches. A typical SST chromatogram is shown in Figure  3B with Rs 15.9 and 19.1 between D-PE2I (region 1)/FE-PE2I and FE-PE2I /Prec-PE2I (region 2), respectively.

This quality control is performed pre-release on all batches.

The non-radioactive impurities in the product formulation can potentially compete in binding with the biological target with our desired radiotracer. In addition, due to some toxicity concerns measuring the exact amount mass of injected product (the mass is important for further molar activity calculation) into the subjects is an important factor. Therefore, developing a sensitive and reliable HPLC method for QC is crucial. The HPLC analysis was performed utilizing an Agilent 1260, mobile phase A: TFA 0.1% and B: Acetonitrile, using the following gradient; 0–1 min A:60 and B:40, 1 to 8 min A:20 and B:80, 8–10 min A:80 and B:20, 10–15 min A:80 and B:20, flow = 0.5 mL/min, column; Poroshell 120 EC C-18, 3 × 150 mm, i.d. 2.7 µm column, injection volume = 50 µL, λ = 220 nm.

#### Radiochemical impurity (impurity B)

**Fig. 4:**
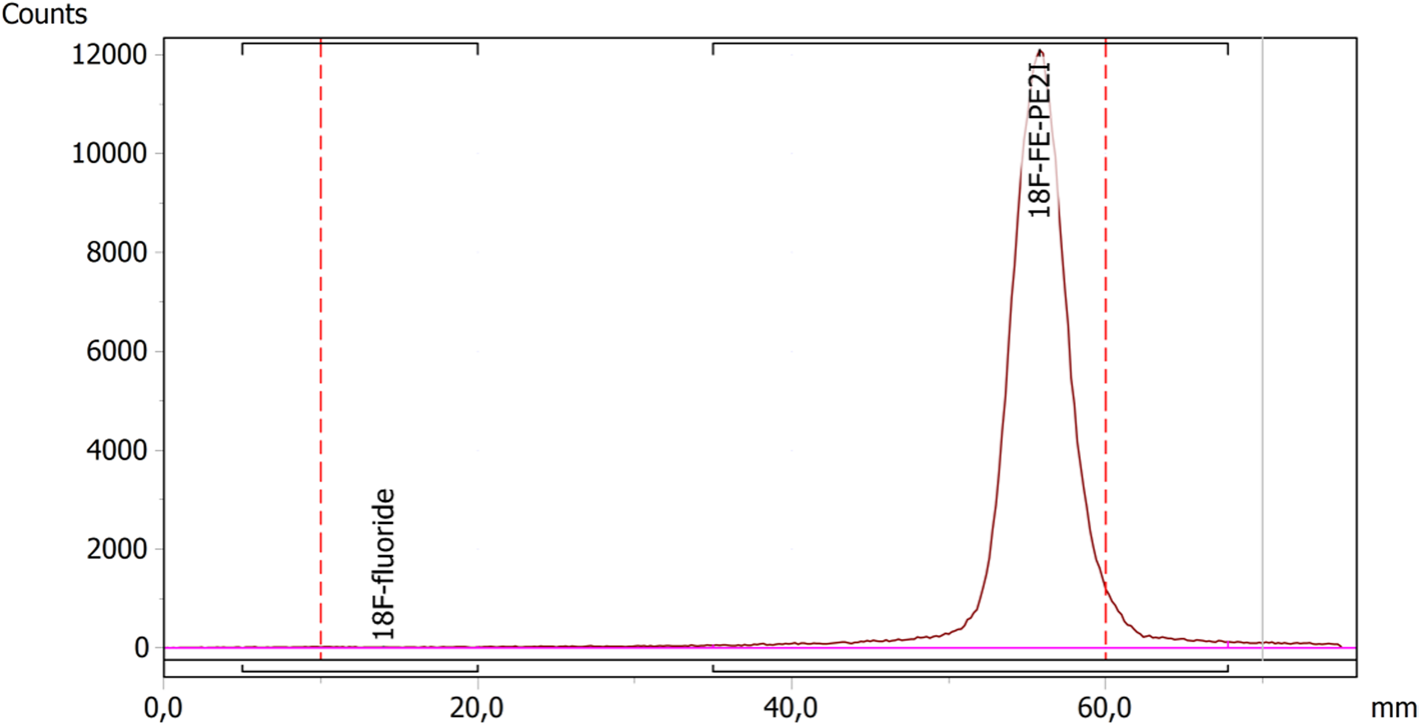
The TLC radio-chromatogram demonstrating the separation of [^18^F]fluoride and [^18^F]FE-PE2I. The retardation factor (R_f_) for [^18^F]fluoride was 0–0.1

A Thin Layer Chromatography (TLC) analysis using a radioactivity detector has been performed to determine the radiochemical impurity percentage of [^18^F]fluoride in the product (Fig. 4). The validated TLC method is already used for clinically approved radiopharmaceutical [^18^F]FE-PE2I, produced at the PET/SPECT center, Umea University Hospital. We verified the method specificity and suitability at our site by performing a single method validation test. The following acceptance criteria are fulfilled: the radioactivity of [^18^F]fluoride was ≤ 5% of the total radioactivity to be released; retardation factors (Rf) for [^18^F]fluoride = 0–0.1 and for [^18^F]FE-PE2I = 0.7–0.9; the peak corresponding to [^18^F]fluoride was completely separated from the peak, corresponding to [^18^F]FE-PE2I and a [^18^F]FE-PE2I product spiked with 5% [^18^F]fluoride. This quality control is performed pre-release on all batches.

#### Total radiochemical purity

The radiochemical product identity is determined by comparison of a sample from the formulated [^18^F]FE-PE2I solution with a reference solution of [^19^F]FE-PE2I pre-analyzed using an HPLC. The eluent is monitored by a UV detector and a radiation detector placed in series. Based on the previous results, the total radiochemical purity, RCP_Tot_ of [^18^F]FE-PE2I Solution for Injection is determined by HPLC and radio-TLC following the formula in-below:

$$RCP_{Tot} = \left( {100 - B} \right) \times T$$

B: Free radioactive [^18^F]fluoride (%) analyzed using radio-TLC analysis.

T: Proportion of the radioactivity due to [^18^F]FE-PE2I using the HPLC analysis.

This quality control is performed pre-release on all batches.

#### Residual Kryptofix 222

The limit specification for residual Kryptofix 222 concentration in [^18^F]FE-PE2I Solution for Injection is < 0.14 mg/mL, calculated according to formula 2.2 mg/V (European Pharmacopoeia Ph.Eur), where V is the maximum recommended injection volume of 15 mL. Kryptofix content is determined in [^18^F]FE-PE2I Solution for Injection as a color spot test based on the standard method for determination of the Kryptofix content in [^18^F]FDG (European Pharmacopoeia, 8.0. EDQM, Strasbourg, France, 2014). The method was modified, since the presence of sodium ascorbate, an antioxidant stabilizer, in the [^18^F]FE-PE2I matrix solution may yield false-negative results. A H_2_O_2_ solution can be added to overcome this issue. Dilution factor in the range of 1–150 was investigated and the results showed that the most suitable dilution factor was 100. In addition, the method showed a proper linearity in the range of 0.025–0.3 mg/mL. Therefore, the dilution factor 100 and the limit specificication for Kryptofix < 0.14 mg/mL were used for all validation and routine batches. This quality control is performed pre-release on all batches.

#### Bacterial endotoxins

Bacterial endotoxins content is determined using the chromogenic kinetic methodology on Endosafe® Nextgen-PTS Kinetic Reader using Test Cartridge PTS2005F. The endotoxins limit of [^18^F]FE-PE2I Solution for Injection is 11.5 EU/mL, calculated according to formula 175 EU/V, where V is the maximum recommended injection volume of 15 mL. This quality control is performed pre-release on all batches.

#### Residual solvents: ethanol, acetonitrile and DMSO

**Fig. 5:**
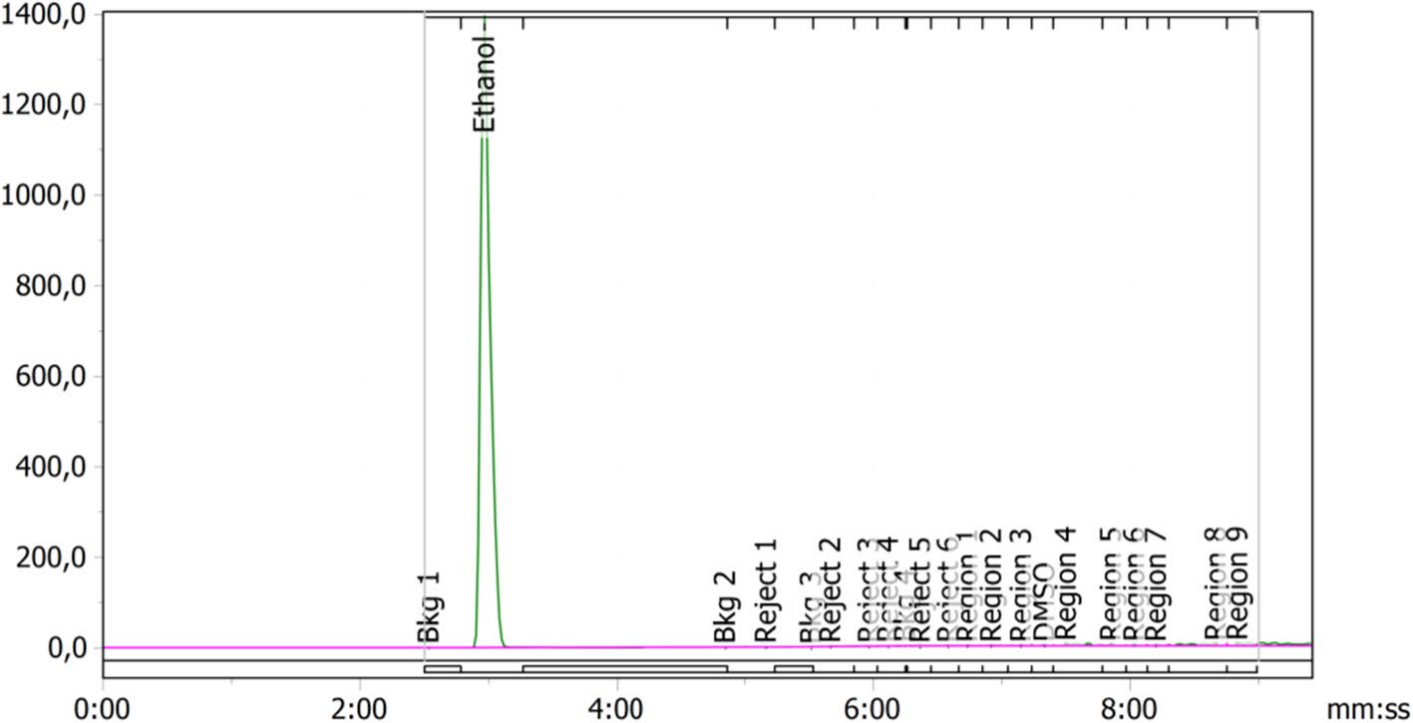
GC chromatogram showing a peak of ethanol (44.1 mg/mL). No traces of DMSO and acetonitrile

The specifications for residual solvents are based on a maximum injected volume of 15 mL. Ethanol concentration and residual acetonitrile and DMSO content are determined via gas chromatography. A SST for GC containing a mixture of ethanol (80.0 mg/mL), acetonitrile (0.27 mg/mL) and DMSO (3.3 mg/mL) was performed, followed by a blank injection of water (18 MOhm water) prior to the QC sample analysis [^18^F]FE-PE2I Solution for Injection. A typical GC chromatogram of [^18^F]FE-PE2I product is shown in Fig. 5. This quality control is performed on all batches; the product may be released before completion of this test.

#### Sterility

Sterility is determined by direct inoculation according to Ph. Eur. This quality control is performed post-release on validation batches and on every 10th clinical batch or every 3rd month.

#### Radionuclidic identity

Radionuclidic identity is confirmed by comparing the half-life of the product with that of fluoride-18 (105–115 min). The half-life is calculated by repeated measurements of product radioactivity using a dose calibrator (CRC-55TR, Capintec). This measurement is performed on the validation and verification batches only.

#### Radionuclidic purity (RNP)

Radionuclidic purity is not analyzed on the product, instead we based RNP of ^18^F-fluoride. In addition, the RNP of ^18^F-fluoride is periodically analyzed. The rationale for it, is that the cyclotron targets generating ^18^F-fluoride have previously been verified in the production of [^18^F]FDG, also no radionuclides can be produced due to irradiation after ^18^F-fluoride leaving cyclotron targets. Therefore, additional analysis of radionuclidic purity is deemed unnecessary for the production of [^18^F]FE-PE2I. Radionuclidic purity is instead analyzed on ^18^F-fluoride after maintenance or after replacement of cyclotron targets.

#### Radiochemical stability

The radiochemical purity of [^18^F]FE-PE2I Solution for Injection was well over specified 93% for at least 6 h when stored at room temperature. Stability data are presented in Table 3.

**Table 3:** Batch analysis for four validation batches of [^18^F]FE-PE2I Solution for Injection (each batch was dispensed into vials)

Test attributes	Product specification	PV1	PV2	PV3	Microbiological worst-case scenario (Bioburden)
Activity concentration	50–1000 MBq/mL	400 MBq/mL	600 MBq/mL	500 MBq/mL	1000 MBq/mL
Appearance	Clear or slightly yellow. Free of particles	Clear or slightly yellow Free of particles. (Both vials)	Clear or slightly yellow Free of particles. (Both vials)	Clear or slightly yellow Free of particles (Both vials)	Clear or slightly yellow Free of particles (Both vials)
pH	4.5–8.0	6.5 (vial 1) 6.5 (vial 2)	6.5 (vial 1) 6.5 (vial 2)	6.5 (vial 1) 6.5 (vial 2)	6.5 (vial 1) 6.5 (vial 2)
Product identity [^18^F]FE-PE2I	[Rt_RD_ – Rt_UV_|< 60 s	6 s (vial 1) 5 s (vial 2)	3 s (vial 1) 2 s (vial 2)	4 s (vial 1) 4 s (vial 2)	3 s (vial 1) 4 s (vial 2)
Chemical purity FE-PE2I	Mass limit ≤ 5 µg/patient dose	≤ 5 µg (both vials)	≤ 5 µg (both vials)	≤ 5 µg (both vials)	≤ 5 µg (both vials)
Chemical impurities	Mass limit ≤ 5 µg per patient dose	≤ 5 µg (both vials)	≤ 5 µg (both vials)	≤ 5 µg (both vials)	≤ 5 µg (both vials)
Radiochemical impurity Impurity = B [^18^F]fluoride	≤ 5%	0% (Both vials)	1% (vial 1) 0% (vial 2)	1% (Both vials)	1% (vial 1) 0% (vial 2)
Total radiochemical purity [^18^F]FE-PE2I RCP_Tot_ = (100 – B) × T	≥ 93%	96% (Both vials)	98% (vial 1) 97% (vial 2)	97% (Both vials)	95% (vial 1) 96% (vial 2)
Mass limit	≤ 5 µg FE-PE2I and ≤ 5 µg impurities per 200 MBq (patient dose)	≤ 5 µg FE-PE2I and ≤ 5 µg impurities	≤ 5 µg FE-PE2I and ≤ 5 µg impurities	≤ 5 µg FE-PE2I and ≤ 5 µg impurities	≤ 5 µg FE-PE2I and ≤ 5 µg impurities
Residual Kryptofix 222 content	< 0.14 mg/mL	< 0.14 mg/mL (Both vials)	< 0.14 mg/mL (Both vials)	< 0.14 mg/mL (Both vials)	< 0.14 mg/mL (Both vials)
Filter integrity vial 1	≥ 3.5 bar	4.4	4.3	4.4	Bioburden
Filter integrity vial 2	≥ 3.5 bar	4.4	4.3	4.4	Bioburden
Bacterial endotoxins	< 11.5 IU/mL	< 5 EU/mL (both vials)	< 5 EU/mL (both vials)	< 5 EU/mL (both vials)	< 5 EU/mL (Both vials)
Ethanol content	< 80 mg/mL	44 mg/mL (vial 1) 43 mg/mL (vial 2)	66 mg/mL (vial 1) 67 mg/mL (vial 2)	68 mg/mL (vial 1) 69 mg/mL (vial 2)	73 mg/mL (vial 1) 76 mg/mL (vial 2)
Acetonitrile	< 0.27 mg/mL	0.00 mg/mL (both vials)	0.00 mg/mL (both vials)	0.00 mg/mL (both vials)	0.00 mg/mL (vial 1) 0.09 mg/mL (vial 2)
DMSO	< 3.3 mg/mL	< 3.3 mg/mL (both vials)	< 3.3 mg/mL (both vials)	< 3.3 mg/mL (both vials)	< 3.3 mg/mL (both vials)
Sterility	Sterile, 0 CFU	Sterile (both vials)	Sterile (both vials)	Sterile (both vials)	Sterile (both vials)
Radionuclidic identity Half-life [^68^ Ga]	105–115 min	110 min (vial 1) 109 min (vial 2)	108 min (vial 1) 108 min (vial 2)	111 min (vial 1) 110 min (vial 2)	111 min (vial 1) 110 min (vial 2)
Radiochemical stability	RCP_Tot_ ≥ 93% up to 6 h EOS	96% (Both vials)	96% (Both vials)	97% (Both vials)	93% (Both vials)
Shelf-life	Batch specific, calculated time where a patient dose exceeds mass limit	6 h	5 h 42 min	5 h 1 min	6 h

*B:* percentage of radioactivity due to impurity [^18^F]fluoride in TLC analysis, *T:* proportion of the radioactivity due to [^18^F]FE-PE2I in the HPLC analysis

## Discussion

Radiosynthesis of [^18^F]FE-PE2I was automated using a commercial radiofluorination module (GE TRACERLab FX2 N), specifically designed for fluoride-18 radiolabeling with an HPLC purification system. We adapted the previously semi-automated protocol reported by Stepanov et al. (2012) to accommodate the commercial radiosynthesis unit, as well as establish quality control procedures that would satisfy the EMA regulatory requirements for GMP production and human PET imaging studies. Radiolabeling was performed in a single-step by a nucleophilic substitution reaction of the tosylethyl-PE2I precursor compound using azeotropically dried potassium cryptand [^18^F]fluoride complex ([^18^F]KF/K_2.2.2_) dissolved in dimethyl sulfoxide. No degradation of the precursor has been observed although a harsh K_2_CO_3_/K_222_ eluent has been used. Therefore, the condition using K_2_CO_3_/K_222_ as eluent seems to be optimal to proceed with.

In the current study, after the completed reaction (140 °C for 150 s), the crude product was diluted with a solution mixture containing sodium ascorbate to prevent possible radiolysis in the reaction vial. Further purification steps have been performed using a semi-preparative HPLC with an ACE 5 C18-HL column and a mixture of water, acetonitrile, trifluoracetic acid, and sodium ascorbate (0.5 mg/mL) as the eluent. The desired fraction was collected and diluted with 5 mg/mL sodium ascorbate in sterile water and finally reformulated using SPE to produce [^18^F]FE-PE2I in a reproducible decay-corrected radiochemical yield of 39 ± 8% (n = 4, relative [^18^F]F^−^ delivered to the module).

The production scaling of [^18^F]FE-PE2I might be challenging due to the use of a high starting activity from the cyclotron that enhance radiolysis occurring mostly during labeling, purification and formulation steps resulting in low RCY. Likewise, the sensitivity of the precursor facing strong base can lead to its decomposition, which can have an influence on the RCY. However, [^18^F]FE-PE2I was produced at Karolinska University Hospital with a starting activity of 50–83 GBq using the conventional K_2_CO_3_/K_222_ elution method. The labeling was performed at 140 °C for 150 s followed by the purification and formulation steps using sodium ascorbate leading to a good RCY.

It is important to note that during the synthesis procedure development, it was found that the addition of sodium ascorbate to the crude product, purification and reformulation steps as well as to the formulated product was necessary to obtain a product with high stability and purity. Removing sodium ascorbate in any part of the procedure resulted in rapid decomposition, likely attributed to radiolysis. Nonetheless, using the above-described conditions, [^18^F]FE PE2I was obtained in high radiochemical purity (> 95%) and a molar activity (Am) of 925.3 GBq/µmol (250 Ci/µmol) at the end of synthesis. The overall synthesis time was 70 min including formulation. Although radioactive losses could likely be minimized by further optimization of the fluorination, (10–15 GBq, 270–405 mCi) were prepared in a form suitable for human use (Table 3).

### Clinical considerations

After Alzheimer´s disease, Parkinson’s disorder is the second most frequent neurodegenerative condition, implying a significant impact on the quality of patients and their family’s life, cost of care and work capacity. Normally, a small and slow dopaminergic reduction occurs in ordinary aging people without causing any symptoms. Dopamine deficiency in the parkinsonian brain is pronounced, emerges much faster and the symptoms are evident. Both PD and APS syndromes show decreased presynaptic neuronal degeneration. The motor symptoms develop gradually and become noticeable in the later phase of diseases when the degradation of dopaminergic neurons is about 50–80% (Simon et al. 2020). In daily clinical practice, the diagnosis of PD and APS is usually founded on history, physical examination, and some clinical guidelines. Still there are no specific tests for certain diagnosis. Brain imaging modalities such as CT, MRI, SPECT and PET/CT may support the suspicion of Parkinson's disease and rule out other disorders.

Until recently, DaTSCAN was the only imagistic method to evaluate dopaminergic activity in the striatum at Karolinska University Hospital. In September 2022, [^18^F]FE-PE2I was introduced, hoping for a more feasible alternative to DaTSCAN. In a comparative study between DaTSCAN and [^18^F]FE-PE2I PET/CT, Marner et al. (2022), found a coequal sensitivity (0.94) and specificity (1.00) in both Parkinson’s disease and atypical parkinsonism.

**Fig. 6:**
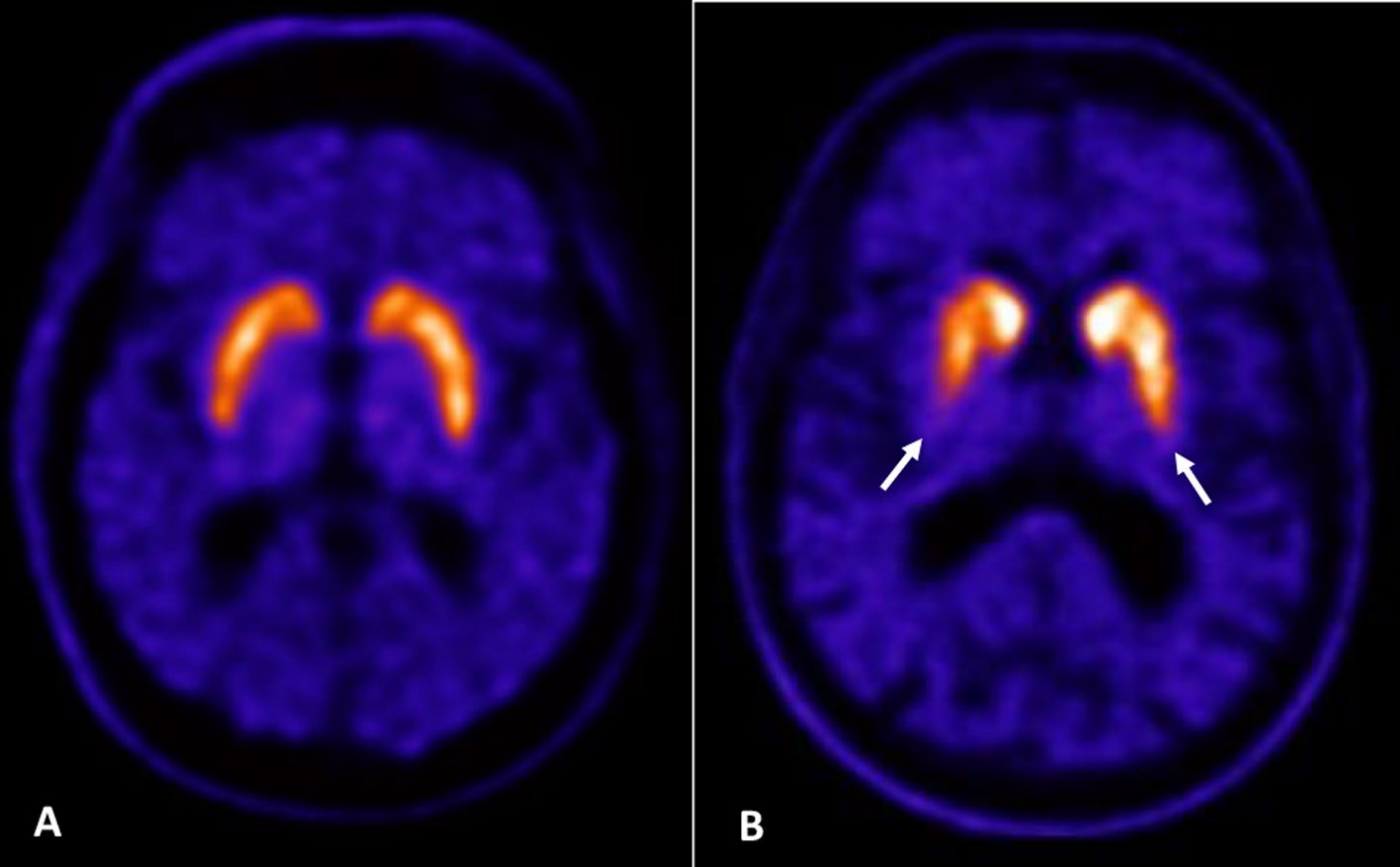
Representative PET/CT images with [^18^F]FE-PE2I at the striatal level investigating Parkinson's disease and parkinsonism

The advantages of using [^18^F]FE-PE2I PET/CT in clinical practice at Karolinska University Hospital are better spatial and temporal resolution of PET/CT compared with SPECT (Fig. 6), reduced time between tracer administration and image acquisition, reduced imaging protocol in static acquisition compared with SPECT (ca 10–15 min compared with 30 min), a more selective and detailed DAT visualization and quantification, and no need for administration of thyroid protecting agents. Moreover, the uptake of [^18^F]FE-PE2I is unaffected by most anti-Parkinsonian medication.

The PET/CT images in Fig. 6 represents: (A) Normal uptake of tracer in both striata in a healthy person. (B) Asymmetric tracer uptake reduction in the putaminae (arrows), with some right-side predominance, and in the right caudate nucleus, with preservation of normal uptake in the left caudate nucleus, in a patient with Parkinson's disease.

## Conclusion

A fully automated synthesis of [^18^F]FE-PE2I was developed on a commercially available radiosynthesis module, GE TRACERLab FX2 N. The decay-corrected radiochemical yield was around 39% and the radiochemical purity was greater than 95%. Overall, the protocol reliably provides a sterile and pyrogen–free GMP-compliant product suitable for clinical use in humans. [^18^F]FE-PE2I can replace or complement DaTSCAN, and at the Karolinska University Hospital, about 300 patients are expected to be scanned with it yearly.

## Data Availability

The datasets used and/or analysed during the current study are available from the corresponding author upon reasonable request. Requests for Tosylethyl-PE2I should be made to PharmaSynth.

## Abbreviations

Am: Molar activity (GBq/μmol)
APS: Atypical parkinsonians
CNS: Central nervous system
CT: Computed tomography
DAT: Dopamine transporter
DMSO: Dimethyl sulfoxide
EOS: End of Synthesis
FE-PE2I: (E)-N-(3-iodoprop-2-enyl)-2β-carbofluoroethoxy-3β-(4’-methyl-phenyl) nortropane
FID: Flame ionization detector
GC: Gas chromatography
GE: General Electrics
GMP: Good manufacture practice
HPGe: High Purity Germanium Radiation detector
HPLC: High-performance liquid chromatography
LOD: Limit of detection
LOQ: Limit of quantification
MRI: Magnetic resonance imaging
NHP: Non-human primate
PD: Parkinson’s disease
PET: Positron emission tomography
PSF: Point spread function
QA: Quality assurance
QP: Quality personnel
Radio-TLC: Radio-thin layer chromatography
RCP: Radiochemical purity (%)
RCP_Tot_: Total radiochemical purity
RCY: Radiochemical yield (%)
RD: Radio-detector
Rt: Retention time
SPECT: Single-photon emission computed tomography
TOF: Time-of-flight
UV: Ultra-violet
